# Malignant hyperthermia: Report on a successful rescue of a case with the highest temperature of 44.2°C

**DOI:** 10.1515/med-2023-0808

**Published:** 2023-10-17

**Authors:** Haiyan Lan, Gongchen Duan, Yunxia Zuo, Tianzheng Lou, Junlong Xu, Chuxiao Shao, Jimin Wu

**Affiliations:** Department of Anesthesiology, Lishui City People’s Hospital, The Sixth Affiliated Hospital of Wenzhou Medical University, Lishui, Zhejiang, 323000, China; Department of Anesthesiology, West China Hospital, Sichuan University, Chengdu, China; Department of Anesthesiology, Lishui City People’s Hospital, The Sixth Affiliated Hospital of Wenzhou Medical University, No. 15, Dazhong Street, Lishui, Zhejiang, 323000, China

**Keywords:** malignant hyperthermia, RYR1 mutation, sevoflurane, case report

## Abstract

Malignant hyperthermia (MH) is an inherited skeletal muscle disorder caused primarily by a genetic mutation, usually in the calcium channel gene of the muscle. This mutation can lead to muscle hypersensitivity to volatile anesthetics (such as sevoflurane) and the depolarizing muscle relaxant succinylcholine, resulting in hyperthermia, muscle stiffness, metabolic disturbances, and other severe physiological reactions. This condition may prove fatal unless it is recognized in its early stages and treatment is administered promptly and aggressively. We report a 13-year-old adolescent who underwent laparoscopic appendectomy and developed MH after the use of inhalational anesthetics, manifested by unremitting hyperthermia with a maximum temperature of 44.2°C, muscle rigidity, tachycardia, hypercapnia; and malignant arrhythmias, cardiogenic shock, hyperkalemia, metabolic, and respiratory acidosis. After early and timely recognition, multidisciplinary management and administration of dantrolene, the case was successfully treated. Exome sequencing revealed a point mutation (amino acid change) on the RYR1 gene: c.12700G>C(p.Val4234Leu). Due to the lack of ready-made dantrolene in our hospital, the patient in this case received dantrolene treatment only 6 h after the first observation of high body temperature. We review the development of the disease and summarize the success of treatment and what can be done to improve the chances of saving the patient’s life if dantrolene is not available in time.

## 1 Introduction

Malignant hyperthermia (MH) is a relatively rare familial genetic muscle disease. In most patients, MH is triggered by conventional inhalation anesthetics or depolarizing muscle relaxants (succinylcholine), typically resulting in systemic muscle rigidity, an abnormally rapid increase in body temperature, and hypermetabolic state. Although the incidence of MH is low, the disease progresses rapidly and has a high mortality rate if not diagnosed and treated promptly [1]. The exact incidence of MH is unknown, but is estimated at 1:10,000–1:250,000 of anesthetic procedures, and children seem to be more susceptible than adults [2]. The mortality of MH is very high, and early diagnosis and treatment are very important.

In May 2022, we successfully treated a patient with fulminant MH, when dantrolene was not available in time, and herein summarize the case.

## 2 Case presentation

A healthy 13-year-old Chinese (Han ethnicity) boy presented for an emergency laparoscopic appendicectomy weighing 46 kg and 160 cm tall with an American Society of Anesthesiologists class I. He had a history of attention-deficit/hyperactivity disorder and premature birth (1 month premature). Prior to surgery he was clinically stable with a febrile temperature of 38.1°C and heart rate (HR) was 117 beats/min.

The patient was admitted to the operating room with the following preoperative vital signs: noninvasive blood pressure of 89/48 mmHg, HR of 85 beats/min, and pulse oxygen saturation of 99%. General anesthesia was induced with midazolam (2 mg), propofol (100 mg), sufentanil (15 µg), and cisatracurium (10 mg) followed by endotracheal intubation. Mechanical ventilation was set up and the end-tidal carbon dioxide (PetCO_2_) and nasopharyngeal temperature (NT) were continuously measured. The airway pressure was 14 mmHg, PetCO_2_ was 35 mmHg, and temperature was 37.3°C. Anesthesia was maintained with sevoflurane inhalation, propofol infusion, and remifentanil infusion.

One hour after the start of the procedure, the patient’s NT rose to 37.5°C, PetCO_2_ rose to 48 mmHg, HR rose to 100 beats/min, airway pressure reached 25 mmHg, and stiffness of the neck muscles developed. In the subsequent 15 min, despite making several adjustments to increase minute ventilation, the end-expiratory carbon dioxide (PetCO_2_) continued to rise from 48 to 81 mmHg. At the same time, his temperature rose to 38.5°C, his HR increased to 120 beats/min, and he developed mixed metabolic and respiratory acidosis on arterial blood gas (ABG) analysis (Table 1). The clinical diagnosis of MH was suspected. Sevoflurane inhalation was immediately stopped, the sodium lime and breathing circuit were replaced, and hyperventilated with 100% oxygen, surgeons were told to end the operation as soon as possible and the hospital crisis management system was activated.

**Table 1:** Intraoperative body temperature and ABG analysis results

Time	Body temperature (℃)	pH value	PCO_2_ (mmHg)	K^+^ (mmol/L)	Lac (mmol/L)	HCO_3_^−^ (mmol/L)	Blood sugar (mmol/L)	BE (mmol/L)
8:23	39.4	7.015	100.1	4.59	2.76	25.0	6.3	−8.3
8:56	44.2	6.974	118.9	6.30	6.14	27.0	7.6	−7.3
9:08	42.7	6.990	76.5	6.14	9.49	18.0	6.5	−13.9
9:19	41.6	6.770	134.0	6.03	8.89	19.0	6.3	−17.5
9:35	40.2	7.089	73.5	5.80	9.69	21.7	6.3	−8.6
9:59	38.1	7.247	45.2	4.62	9.37	19.2	5.2	−7.7
10:45	36.8	7.003	88.3	3.86	7.25	21.4	7.6	−11.2
11:20	36.2	7.031	87.3	3.76	6.53	22.6	5.6	−9.7
11:57	36.4	7.108	69.0	3.83	6.11	21.3	4.3	−9.2

Note: General anesthesia was induced at 06:45, sevoflurane started to inhale at 06:55, the procedure was started at 07:15.

Considering the unavailability of dantrolene in our hospital, a multidisciplinary evaluation was performed, and renal replacement therapy was immediately initiated. Symptomatic treatment was carried out, including: (1) cooling therapy (brain protection with ice caps and alcohol wipe downs, intravenous infusion of cold saline, ice bags placed on the neck, armpits, popliteal fossa, and groin, and cold saline rinses in the abdominal cavity); (2) correction of acidosis (hyperventilation and intravenous administration of sodium bicarbonate); (3) correction of electrolyte disturbances, mainly hyperkalemia (calcium, sodium bicarbonate, furosemide diuresis, glucose, and insulin) and monitoring of blood glucose; (4) appropriate use of vasoactive drugs (norepinephrine and epinephrine) to maintain hemodynamic stability; and (5) administration of high-dose methylprednisolone shock therapy (500 mg) to alleviate systemic inflammation. Intravenous administration of heparin (2 mg) was also given to prevent disseminated intravascular coagulation (DIC). During this period, the body temperature continued to rise, reaching a maximum of 44.2°C and PetCO_2_ increased to 143 mmHg. Within 10 min after starting continuous renal replacement therapy (CRRT), his temperature began to drop and recovered to 38.1°C. PetCO_2_ levels also decreased to 81 mmHg and acidosis improved (Table 1). After stabilization, the patients were transferred to the intensive care unit (ICU) for further treatment.

After obtaining dantrolene, the initial dose of 2 mg/kg (Li Zhu De Le Pharmaceutical, China) was immediately administered, followed by intermittent bolus of 1 mg/kg every 4–6 h. Intravenous dantrolene was continued for another 2 days, during which time he did not have any signs of recrudescence, renal impairment, or other overt complications of MH. During his ICU stay, he initially developed rhabdomyolysis, resulting in elevated creatine kinase (CK) and myoglobinuria. His CK and myoglobinuria levels both decreased after peaking on postoperative day (POD) 5 (Table 2). Liver function tests, complete blood count, and cardiac troponin levels gradually returned to normal after appropriate treatment.

**Table 2:** Changes in CK and myoglobin

Index	Before anesthesia	After anesthesia 4 h	POD2	POD3	POD4	POD5	POD8	POD11
CK (U/L)	222	2,500	79,378	87,020	>80,000	120,450	12,818	8,478
Myoglobin (μg/L)	Not measured	>10,000	>10,000	>10,000	>10,000	>10,000	7,018	3,521

POD: postoperative day.

**Figure 1:**
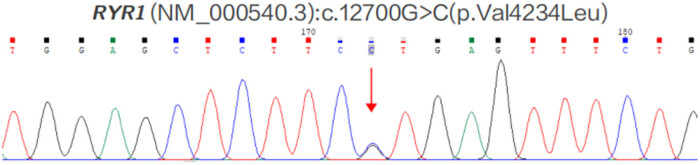
Exome sequencing revealed a point mutation (amino acid change) on the *RYR1* gene: c.12700G>C(p.Val4234Leu).


With the consent of the patient’s family, the patient underwent whole-exome sequencing. The sequencing revealed a point mutation (amino acid change) on the RYR1 gene: c.12700G>C(p.Val4234Leu) (Figure 1). However, the patient’s parents declined further testing to confirm the suspicion of MH.

**Consent for publication:** The authors have obtained the patient’s father written informed consent for print and electronic publication of this case report.

## 3 Discussion

MH is an abnormal metabolic hyperactivation of skeletal muscle spasms that is induced by exposure to inhaled anesthetics and depolarizing muscle relaxants. As medical treatment has improved, the number of operations performed under general anesthesia has increased annually; additionally, with the progress of anesthesia monitoring methods and the improvement of doctors’ knowledge of MH, the number of reported cases of MH has increased in recent years [3]. The current gold standard for diagnosing MH is the caffeine-halothane contracture test. However, this test is not widely available or commonly used. Nowadays, the identification of a pathogenic mutation is also considered diagnostic for MH. A clinical diagnosis of MH can be assisted by calculating the Clinical Grading Score, in which a score of 50 points and grade of 6 almost certainly indicates MH [1,4]. Our patient had a score of 83 points (systemic muscle stiffness, 15 points; CK of >10,000 U/L without succinylcholine after anesthesia, 15 points; PetCO_2_ of >55 mmHg with sufficient minute ventilation, 15 points; abnormally rapid increase in perioperative body temperature, 15 points; abnormal tachycardia, 3 points; the base excess more negative than −8 on ABG analysis, 10 points; pH of <7.25 on ABG analysis, 10 points).

The patient underwent whole-exon sequencing to identify the mutations in the RYR1 gene. This variation locus has been classified in the ClinVar database as suspected pathogenic, and it has been detected in multiple patients with MH episodes or individuals with a family history. But this is the first time that the point mutation has been reported in the general East Asian population, and the prediction result of both REVEL software and ClinPred software was “harmful” (score of 0.890 and 0.9740, respectively). Studies have confirmed that ryanodine receptor type 1 gene (*RYR1*) abnormalities are the molecular basis for MH in most patients [5–7]. Mutation in the *RYR1* gene results in abnormalities in the sarcoplasmic reticulum of skeletal muscle, leading to a high calcium concentration when the patients touched trigger factor such as inhalation anesthetics, followed by continuous tonic contraction of skeletal muscle [8]. MH is an autosomal dominant disorder, and the offspring of MH patients have a 50% risk of developing this disease. Consequently, it is important to fully inform individuals that test results may impact their access to health insurance, employment opportunities, and even marriage [9]. Dantrolene, as a special treatment medication for MH, can effectively inhibit the release of calcium ions in the sarcoplasmic reticulum, thereby inducing muscle relaxation [10]. Early use of dantrolene can reduce perioperative mortality of MH. However, the drug is expensive and difficult to preserve, and only a few hospitals have its stocks in China. Therefore, this reminds us that patients using inhalation anesthesia or depolarizing muscle relaxants should continuously monitor their body temperature and timely differential diagnosis and early treatment should be performed.

Typically, CK peaks after 72 h. However, there was a delay in peak in this case, which may be related to compartment syndrome, and the fact that the patient had rhabdomyolysis symptoms such as systemic muscle rigidity and elevated CK does not rule out compartment syndrome. Moreover, the patient’s CK peak exceeded 100,000 U/L, similar to those previously reported in some patients with MH.

Our experience illustrates several important issues in MH management, if dantrolene is not immediately available. (1) Improved monitoring is very important for the early detection and diagnosis of MH, and early diagnosis and early treatment are the basis of successful rescue of MH. (2) It is critically important to quickly convene the whole hospital for multidisciplinary joint diagnosis and treatment. (3) It is important to use a variety of cooling techniques. In this case, a variety of cooling methods are used to bring the body temperature down below the danger range as quickly as possible. Extracorporeal circulation can be used for cooling if necessary. (4) CRRT is beneficial to maintain internal environmental homeostasis, correct electrolyte disturbance and acidosis, maintain hemodynamic stability, and more effectively remove myoglobin and part of inflammatory mediators by means of convection and adsorption. It can lower the body temperature of patients with hyperthermia by lowering the temperature of replacement fluid, and provide nutritional support under high metabolic state. Prevent and treat renal failure. (5) Early application of heparin therapy reduces the consumption of coagulation factors and platelets by DIC. Ensure hemodynamic stability and give adrenocorticosteroids and diuretics if necessary. (6) Extracorporeal membrane oxygenation (ECMO) may be used as a last resort in patients with sustained cardiac arrest. (7) MH is rare, and many hospitals have insufficient experience in rescue. Timely and effective contact with anesthesia experts in other hospitals to guide the treatment is also important for successful rescue.

## 4 Conclusion

The characteristics of MH are rapid onset, high mortality rate, and numerous complications. Dantrolene can greatly improve the prognosis of patients. However, if dantrolene is not routinely available in the hospital, it is necessary to take correct symptomatic treatment measures, including CRRT, lowering body temperature, brain protection, maintaining stable vital signs, ECMO, etc., to save valuable time for obtaining dantrolene. Furthermore, dantrolene should be routinely equipped in every hospital or region whenever possible.

## Abbreviations

ABG: arterial blood gas
CK: creatine kinase
CRRT: continuous renal replacement therapy
DIC: disseminated intravascular coagulation
HR: heart rate
ICU: intensive care unit
MH: malignant hyperthermia
PCO_2_
: partial pressure of carbon dioxide
PetCO_2_
: the end-tidal carbon dioxide
RYR1: ryanodine receptor type 1 gene
